# Helical model of compression and thermal expansion

**DOI:** 10.1038/s41598-023-44467-y

**Published:** 2023-10-13

**Authors:** Sylwia Zięba, Michalina Rusek, Andrzej Katrusiak, Andrzej Gzella, Alina T. Dubis, Andrzej Łapiński

**Affiliations:** 1 Institute of Molecular Physics, Polish Academy of Sciences, Smoluchowskiego 17, 60-179 Poznan, Poland; 2 Faculty of Chemistry, Adam Mickiewicz University, Uniwersytetu Poznańskiego 8, 61-614 Poznan, Poland; 3 Department of Organic Chemistry, Poznan University of Medical Sciences, Grunwaldzka 6, 60-780 Poznan, Poland; 4 Faculty of Chemistry, University of Bialystok, Ciołkowskiego 1K, 15-245 Bialystok, Poland

**Keywords:** Materials science, Physics

## Abstract

A negative linear temperature expansion and a negative linear compressibility were observed for imidazolium benzoate salt. Its strongly anisotropic strain induced by the temperature and pressure changes has been explained by the mechanism of H-bonded helices deformed in the structure. X-ray diffraction and vibrational spectroscopy were used to analyze interactions in the crystal. The Quantum Theory of Atoms in Molecules (QTAiM) approach was applied to analyze the hydrogen bonds and other interactions. In the salt under study, the interactions within the helix are substantially higher in energy than between helices. With decreasing temperature and increasing pressure, the value of the helix pitch increases while the value of the semi-major axis decreases, which results in the negative linear expansion and negative linear compression, respectively.

## Introduction

Negative linear compressibility (NLC) and negative area compressibility (NAC) are rare types of crystal strain in which one or two dimensions expand with increasing hydrostatic pressure^1–3^. In general, NLC is much weaker than conventional materials' typical positive compressibility^4^. Materials that exhibit negative linear compressibility can be used to build, for example, pressure sensors, artificial muscles, or actuators^4^. In most crystals, their dimensions changes are monotonic^5^. Negative thermal expansion (NTE) can also occur in materials where NLC is observed^6,7^. The NTE is a phenomenon in which one, two or three dimensions reduce with increasing temperature^6^. The phenomenon of negative and zero temperature expansion can find application, for example, in thermomechanical sensors, actuators, precision optical mirrors, fiber optic systems, packaging materials for meshes with the suitable light coefficient, high-performance explosive sensors^1,3,8,9^.

Negative temperature expansion can occur in crystals along one, two, or three principal axes. Negative temperature expansion in one direction has been observed in such crystals as 2,6-dimethylaniline monohydrate (BTA-DMA-H_2_O)^10^, nitromethane solvate 18-crown-6^11^, hydrated tryptophylglycine dipeptide (TrpGly-H_2_O)^12^. We can also distinguish a group of crystals in which NTE occurs in two directions: (S,S)-octa-3,5-diyn-2,7-diol^13^, 2,4-dinitroanisole^14^. There is also a group of materials with volumetric negative temperature expansion: Y_2_Mo_3_O_12_^15^, and M^II^Pt^IV^(CN)_6−x_{H_2_O}^16^. There is also a small group of compounds in which we observe near-zero temperature expansion: YbGaGe^17^, (1 − x)PbTiO_3−x_Bi(Mg, Ti)_1/2_O_3_^18^. One way to obtain such materials is to synthesize them from a mixture of compounds exhibiting PTE and NTE^17^. Very rarely are combinations of PTE, NTE, and ZTE in the same material^19^. Such a situation is explained by the “stretching-tilting mechanism”, “hinge mechanism,” or “lattice fence mechanism”^20,21^.

In the literature, however, no single mechanism explains the nature of the NTE phenomenon. From time to time, there are reports describing the explanation of the origin of the anomalous behavior of the material by various mechanisms: e.g., the “transverse vibration” effect^8,22,23^, “scissors” effect^24^, “accordion motion”^25^, “the sliding of layers”^10,25^, “jack motion”^26^, “fencing mechanism”^13,21^, “pedal-wheel motion”^27^. Recently, Cairns and Goodwin^4^ grouped materials exhibiting NCL into four groups depending on the microscopic mechanism responsible for NLC: (a) compounds in which NLC is a consequence of ferroelectric phase transitions, (b) network solids for which correlated polyhedral tilts drive NLC, (c) helical systems, and (c) framework materials with wine-rack, honeycomb, or related topologies, where NLC arises from framework hinging. Ferroelastic instabilities are exhibited by rutile, in which ferroelectric instability is due to the rotation of neighboring columns of octahedra^28^, and zinc cyanide, with symmetry-braking mechanisms, such as a phonon instability^29^. Polyhedral tilting has been observed in the nonlinear optical (NLO) material BiB_3_O_6_; the dominant deformation mechanism under hydrostatic pressure involves correlated tilting of BO_3_ units, which act to hinge the connected borate framework^30^. The most likely mechanism responsible for NLC in the system would involve PO_4_ rotation-driven collapse of the herringbone hydrogen-bonding network^31^. The helix, responsible for NLC, was observed in trigonal polymorphs of elemental selenium and tellurium^32^. A molecular framework mechanism has been described for silver(I) hexacyanocobaltate(III), Ag_3_[Co(CN)_6_]^6^.

In the compounds derived from carboxylic acids and heterocyclic molecules, anomalous temperature expansion^10,20,33^ and negative linear compressibility^7^ were observed. In compounds with negative temperature expansion, negative pressure expansion often occurs^5,7^. A change in pressure from 0.2 to 0.5 GPa causes similar changes in crystal volume as a reduction in temperature from 300 to 100 K^34^. The effects of increased pressure are similar to those observed under conditions of lowering the temperature for organic and metal–organic crystals^35^. As early as the 1980s, it was pointed out that hydrogen bonds can affect the occurrence of anomalous temperature expansion and negative linear compressibility^31^.

The authors of this paper have studied the physicochemical properties of compounds derived from carboxylic acids and heterocyclic molecules^19,36,37^. The attraction of this group of materials lies in the fact that the physical properties are influenced by the hydrogen bonding network in these salts^10,38^. By appropriately selecting substrates in the synthesis, we can control the amount and strength of hydrogen bonds. Our previous structural and spectroscopic studies carried out for (bis)imidazolium terephthalate^19^ showed the occurrence of negative temperature expansion phenomena, which is associated with the N^+^–H⋯O^−^ hydrogen bond network.

The phenomenon of negative temperature expansion was also observed for organic compounds with helix-forming hydrogen bonds: (*S,S*)-octa-3,5-diyn-2,7-diol^13^ and (bis)imidazolium terephthalate^19^. As early as the 1970s, attempts were made to link the occurrence of the helix motif to the phenomenon of negative linear compressibility^32^. From the literature, we know several ring molecules based on which the helical structure was obtained, such as (7)phenylene^39^, oligoamides^39^, or tetrakis(7)helicene^40^. Intramolecular and intermolecular hydrogen bonds play a stabilizing role and influence the organization of molecules in the helix^39^. The type and shape of the helix are influenced by the substituents attached to the aromatic ring and π–π interactions^39,40^. Molecules forming a helical structure are distinguished by hydrogen bonding ability, chirality, hydrophilicity, or hydrophobicity^41^.

In the above work, we propose a helical model, to our knowledge not previously found in the literature, to explain the crystal’s anomalous temperature and pressure behavior. For structural and spectroscopic studies, we chose imidazolium benzoate salt (**BenImi**)^37^, for which a helical structure formed by ions connected by hydrogen bonds N^+^–H···O^−^ is observed.

## Results and discussion

On the basis of X-ray analysis, it was determined that the **BenImi** compound is imidazolium benzoate (formula moiety: C_7_H_5_O_2_^−^, C_3_H_5_N_2_^+^) (Fig. S1), crystallizing in a monoclinic system and space group *P*2_1_/*n* (Tables S1 and S2). The asymmetric part of the unit cell is composed of one anion and one cation molecule.

**Figure 1:**
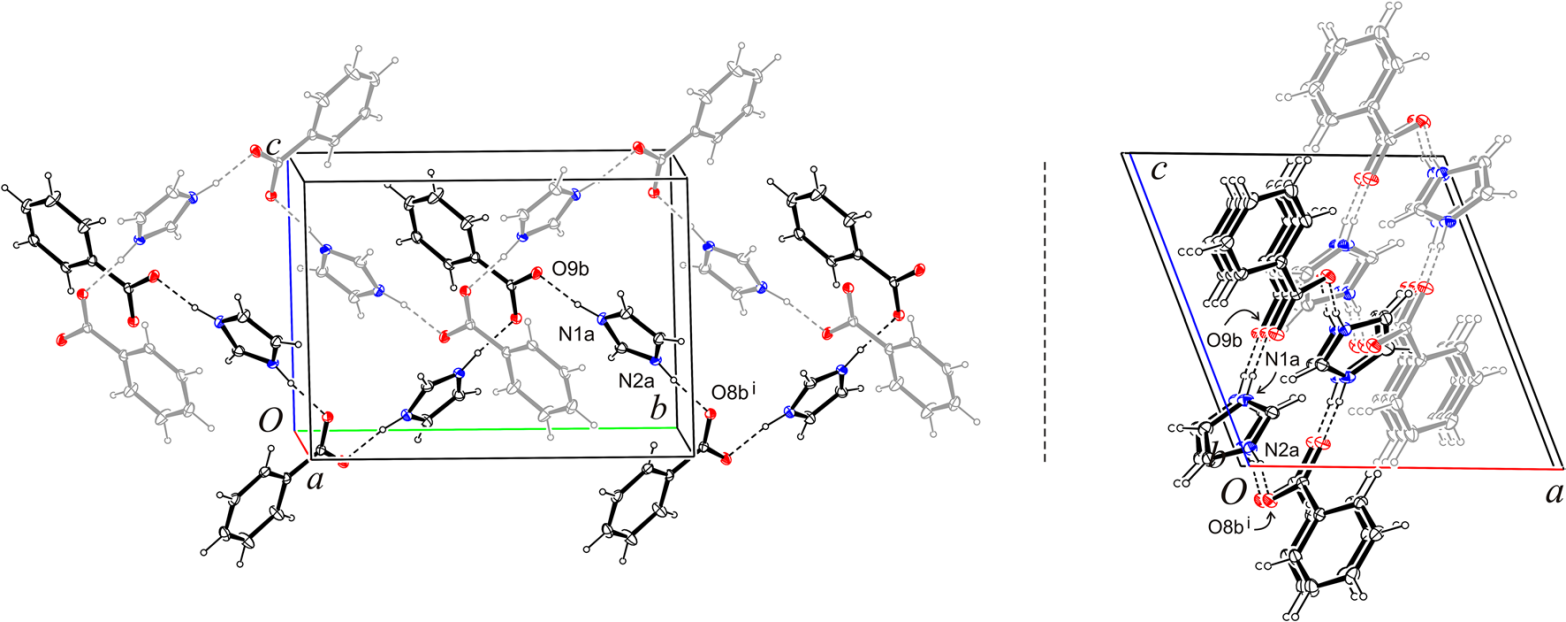
The helical structure in **BenImi** formed by cations and anions connected by N^+^–H···O^−^ hydrogen bonds.

Analysis of the crystal's structure revealed that the molecules of imidazolium cation and benzoate anion, related by 2_1_ axis, are connected by hydrogen bonds N1a–H1a∙∙∙O9b and N2a–H2a∙∙∙O8b^i^ into helix-shaped chains (Fig. 1). Neighboring antiparallel chains are further connected by hydrogen bonds C1a–H1aa∙∙∙O9b^ii^ into beta-harmonic layers parallel to the ($\overline{1}01$) plane (Tables S3 and S4, Fig. S2). Analysis of the layer’s structure showed that the molecules of the benzoate anion form its outer shell, while the molecules of the imidazolium cation the inner ribbing (Fig. S2). Interactions C–H⋯*Cg*1^iii^ were noted between the layers (Fig. S2).

At various temperatures (100, 130, 180, 240, 300 K), X-ray analyses on crystals of imidazolium benzoate were conducted. They demonstrated the stability of the crystal structure in the temperature above range, especially the invariability of its symmetry (vide supra). The situation differs for the unit cell parameters *a*, *b*, *c*, and *β* angle, which undergo observable changes (Table S5). Two of the three parameters of the unit cell, namely *a* and *c*, as well as the *β* angle, exhibit constant elongation (increase) as the temperature rises, whereas the parameter *b* decreases. Despite the different behavior of the parameters *a*, *c* relative to *b*, the volume of the unit cell (*V*) increases from 923.38(10) to 958.27(15) Å^3^ with increasing temperature (from 100 to 300 K) (Table S5).

Positive temperature expansion of crystalline materials is a well-known physical phenomenon^42^. As the temperature increases, we observe an increase in the distance between atoms in the crystal. This behavior leads to an increase in the dimensions of the crystal on a macroscopic scale. The amplitude of temperature expansion depends on the type of interactions between molecules and the packing of the crystal lattice^43^. Anomalous temperature expansion in solids can be interpreted by analyzing the interactions between molecules or ions. It is most often explained by analyzing the strength of the interaction, the asymmetric shape of the potential energy curve, or structural distortions.

In the **BenImi** crystal lattice, ions interact with N^+^–H⋯O^−^ and C‒H⋯O hydrogen bonds. The donor–acceptor distances with temperature change (100/300 K) are 2.60/2.61 Å, 2.65/2.67 Å (for N^+^–H⋯O^−^) and 3.20/3.19 Å (for C‒H⋯O). The following intermolecular hydrogen bonds were found: classical N1a‒H1a⋯O9b and N2a‒H2a⋯O8b^i^ (within helixes) and non-classical C1a‒H1aa⋯O9b hydrogen bonding (between helixes) (Fig. S3). The geometrical parameters of hydrogen bonds: distance of donor hydrogen $d_{{{\text{D}} - {\text{H}}}}$, hydrogen-acceptor $d_{{{\text{H}} - {\text{A}}}} ,$ donor–acceptor $d_{{{\text{D}} - {\text{A}}}} ,$ and hydrogen bond angle ∠D–H···A are summarized in Tables S3 and S4.

Calculations were performed using the quantum theory of atoms in molecules (QTAiM)^44^ and Espinosa's approximation (*E*_HB_ ∼ 1/2 *V*_BCP_)^45^ to determine the hydrogen bonding energies (Fig. S4, Tables S6 and S7). The *V*_BCP_ is the potential electron energy density at the bond critical point (BCP). The growth of electron density at the BCP is related to the increase in hydrogen bond strength. Bond critical points (BCPs) occur for hydrogen bonds: N1a–H1a⋯O9b, N2a–H2a⋯O8b^i^, and C1a–H1a⋯O9b. Intermolecular hydrogen bonds N1a–H1a⋯O9b and N2a–H2a⋯O8b^i^ are *E*_HB_ = − 16.88 kcal·mol^−1^ and − 17.18 kcal·mol^−1^, respectively; they are medium strength. Unconventional hydrogen bond C1a‒H1a⋯O9b is *E*_HB_ = − 1.82 kcal·mol^−1^; it is weak strength. It shows that interactions within a helix are stronger than between helices in the salt under study.

Most compounds in the solid phase expand during heating^1,2^ and pressure reduction^34^. A positive coefficient of temperature expansion and a positive coefficient of volume compressibility characterizes them. With decreasing temperature/increasing pressure, it is possible to observe the geometric contraction of certain compounds^2,17^. We can divide temperature/pressure expansion into volumetric and linear expansion. In the first case, we are talking about the change in volume as a function of temperature/pressure. In the second, we are talking about the change in length; in the direction of the principal axes X1, X2, and X3^13^. The coefficients of volumetric $(\alpha_{V} ,\; K_{V} )$ and linear $(\alpha_{L} , \;K_{L} )$ thermal expansion (α) and compressibility (K) are determined from the Eqs. (1) and (2), respectively^1,10^:

1 $$\alpha_{V} = \frac{\Delta V}{V}\frac{1}{\Delta T},\;\;\;K_{V} = - \frac{1}{V} \cdot \frac{\Delta V}{{\Delta p}},$$

2 $$\alpha_{L} = \frac{\Delta l}{l}\frac{1}{\Delta T},\;\;\;K_{L} = - \frac{1}{l} \cdot \frac{\Delta l}{{\Delta p}}$$

where: ∆V—change in volume of the crystal, ∆T—change in temperature, ∆p—change in pressure, and ∆l—change in length in the X1, X2, or X3 direction.

**Figure 2:**
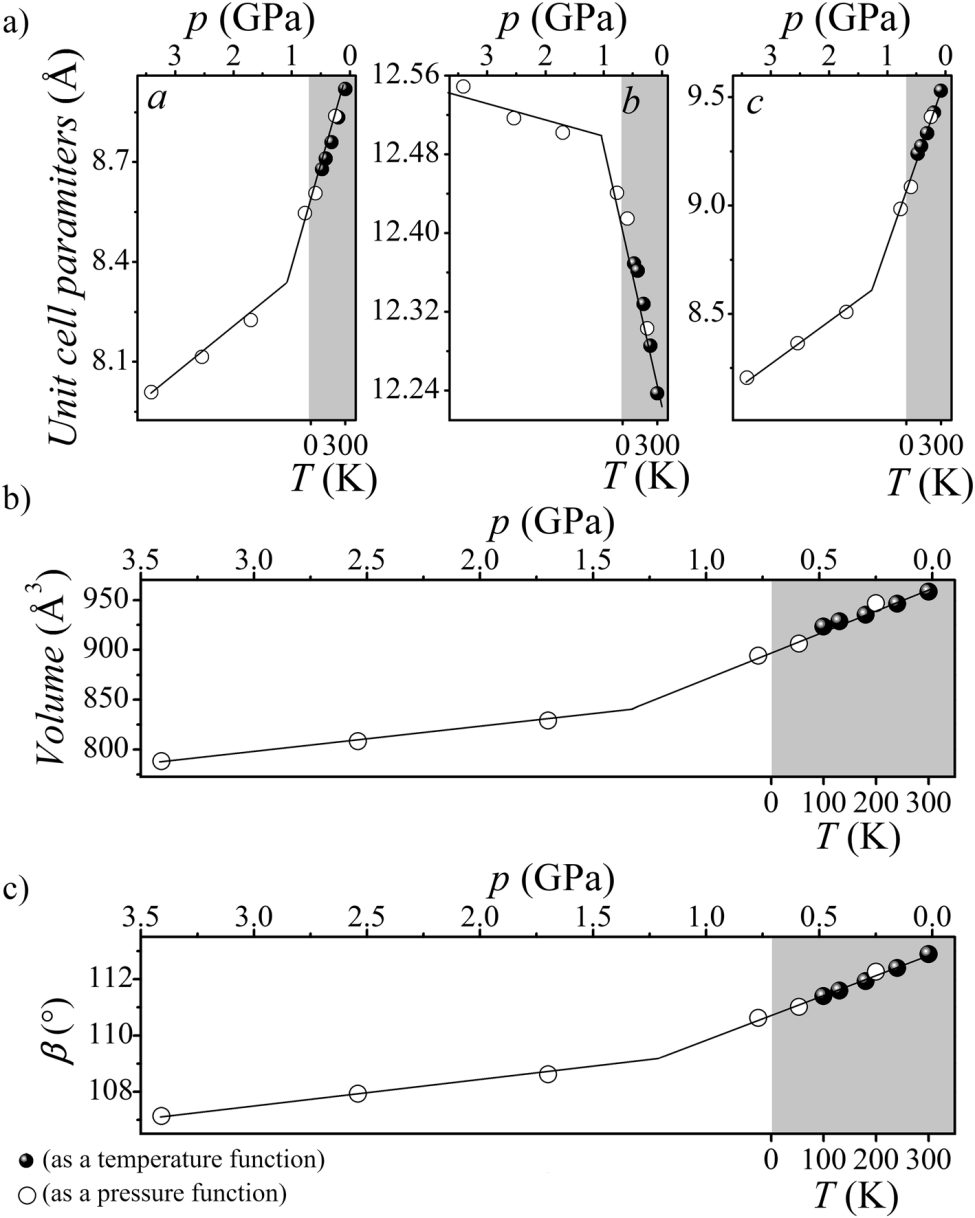
Temperature and pressure dependence of unit cell parameters *a*, *b*, and *c* (**a**), unit cell volume *V* (**b**), and angle *β* (**c**) determined for **BenImi**.

As the temperature decreases, the values of the parameters *a*, *c*, and *V* for imidazolium benzoate get smaller while the parameter *b* increases (Fig. 2, Table S1). The salt behaves similarly with increasing hydrostatic pressure (Fig. 2, Table S2).

**Figure 3:**
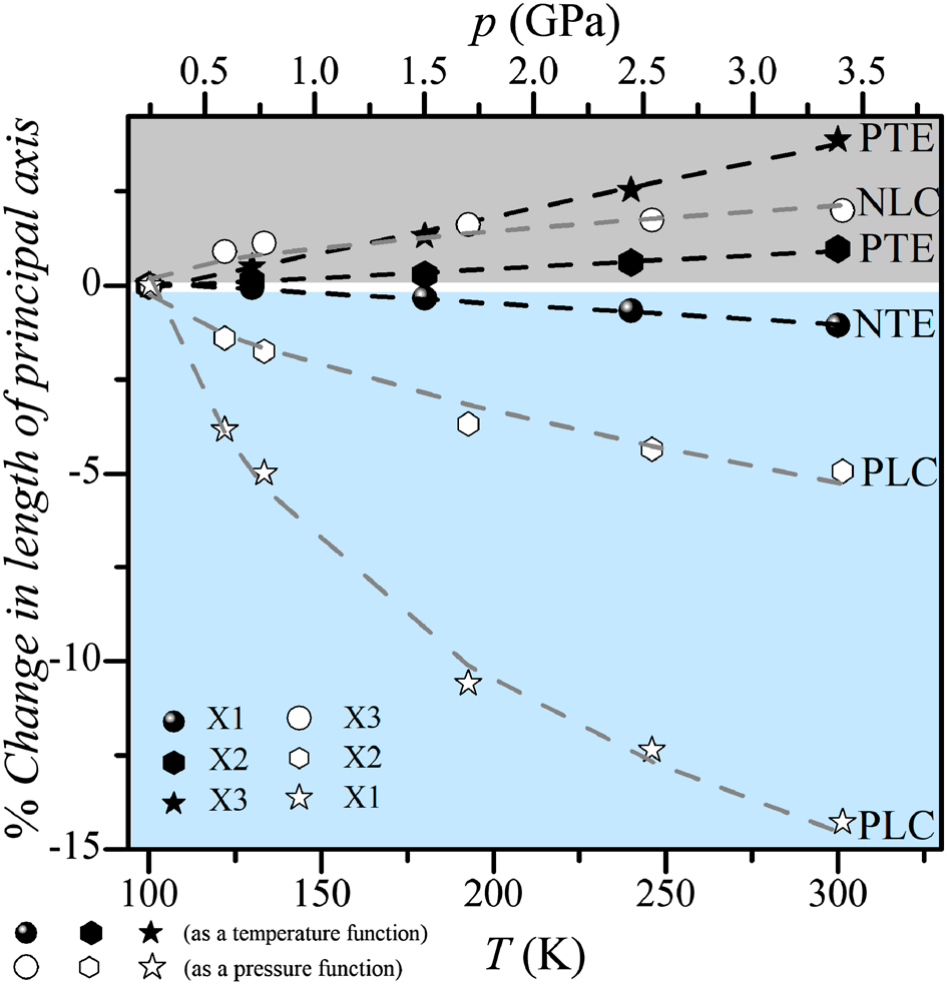
Relative changes in principal axes lengths as a function of temperature and pressure. *PTE* positive temperature expansion, *NTE* negative temperature expansion, *PLC* positive linear compressibility, *NLC* negative linear compressibility.

Using the PASCal program^46^, the principal axes were determined for temperature changes: X1_*T*_ [0, − 1, 0], X2_*T*_ [0.7488, 0, 0.6628], and X3_*T*_ [− 0.6838, 0, 0.7297]. For pressure changes, the following orthogonal set of axes is X1_*p*_ [− 0.4688, 0, 0.8833], X2_*p*_ [0.8226, 0, 0.5687], and X3_*p*_ [0, − 1, 0] (see Fig. 3). The directions X1_*T*_, X2_*T*_, and X3_*T*_ correspond to X3_*p*_, X2_*p*_, and X1_*p*_, respectively. The coefficients of linear temperature expansion are equal to: -54.6(2), 47.8(8) and 193.2(5) 10^–6^·K^−1^ for the X1_*T*_, X2_*T*_ and X3_*T*_ principal directions. The coefficient of volumetric temperature expansion is equal to 187.7(2) 10^–6^·K^−1^. The coefficients of linear compressibility are equal to 25.2(9), 12.2(7), and − 4.2(6) TPa^−1^. The volumetric compressibility coefficient equals 55.4(8) TPa^−1^.

The value of the above coefficients of temperature expansion and compressibility shows that **BenImi** has positive and negative linear temperature expansion and positive volumetric temperature expansion, and positive volume compressibility (Fig. 3). We observe negative temperature expansion and negative compressibility in the X1_T_ and X3_p_ axis and positive temperature expansion and positive linear compressibility in the X2_T_, X3_T_, X1_p,_ and X2_p_.

Analysis of the linear temperature expansion in **BenImi** showed that the interatomic distances along the X2_T_ [0.7488, 0, 0.6628] and X3_T_ [− 0.6838, 0, 0.7297] shorten upon decreasing temperature. Changes in X2 directions are mainly related to C‒H⋯O hydrogen bonds, and in X3, related to changes in the strength of C‒H⋯π interactions. The elongation of interatomic distances in the X1 [0, − 1, 0] direction is predominantly related to the *b* parameter of the unit cell. This direction is parallel to the helical chains formed by cations and anions connected by hydrogen bonds N^+^–H⋯O^−^. With decreasing temperature/increasing pressure, the distances between ions in the X1_T_, X3_p_ direction increase while leading to an decrease in the distance between molecules along the X2_T_, X2_p_ and X3_T_, X1_p_ directions. Based on the structural data analysis, it could be concluded that the imidazolium ion performs a librational motion, which leads to an increase in the distance between the ions (X1_T_/X3_p_ direction).

As temperature and pressure change in the **BenImi** crystal structure, the relative arrangement of molecules changes. The angle between the anion and cation plane (θ) at 300 K equals 65.4°, and for 100 K, it is 60.7°. At 0.25 GPa, it is 63.5°, and at 3.41 GPa, it is 49.2°. The angle θ decreases with decreasing temperature and increasing pressure (Fig. S5). The dihedral angle between the carboxylate group and the phenyl ring changes with temperature. At 100 K, this angle equals 7.59°, and at 300 K, it is 9.16°. The change in this angle is also observed in the pressure function. At 0.15 GPa, it is 8.23°, and at 3.41 GPa, it is 7.25°. The phenyl ring is twisted relative to the carboxylate groups, indicating that the carboxylate groups are rotated with the change in temperature and pressure. It was also observed that as the crystal temperature decreases, the distance between acid anions in **BenImi** increases. The C7a–C9a–N1b–C1b dihedral angle also changes from 18.18° (300 K) to 15.50° (100 K) and from 18.19° (0.25 GPa) to 13.42° (3.41 GPa). The observed changes indicate the rotation of the imidazole ion around the axis, passing through the carbon atoms of the five-membered ring (Fig. S6). Hence, it is to be expected that the librational motion of the imidazole ion affects the dynamics in the transverse direction of hydrogen bonds.

Network vibrations (phonons) significantly affect temperature expansion, especially at low temperatures^2^. When increasing the temperature, longitudinal vibrations lead to an increase in the size of the crystal^22^. Transverse vibrations may cause crystal size reduction with increasing temperature^1,22^. In a helical system, hydrogen bond shortening induced by transverse vibrations can lead to the elongation of the helix and its constriction in the direction perpendicular to its axis^13,33^. Transverse vibrations can be a source of NTE, especially at low temperatures^1,2^.

**Figure 4:**
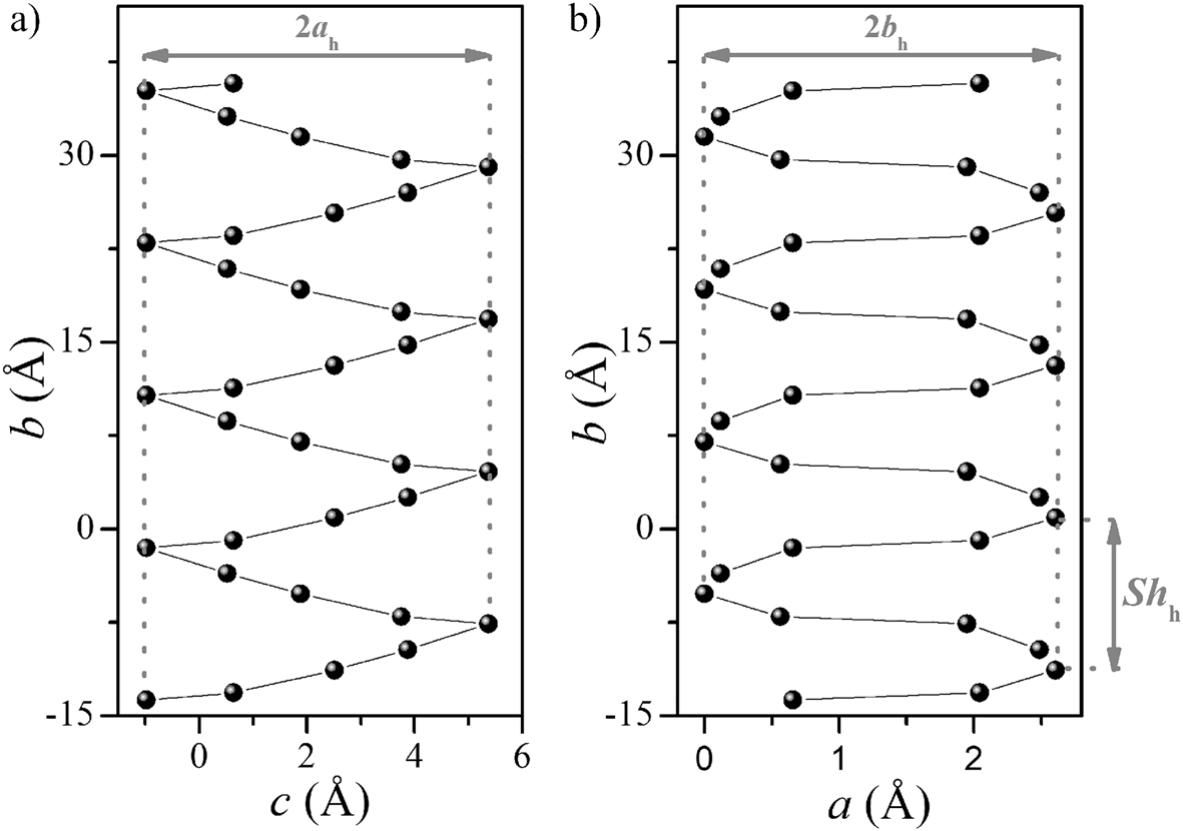
Projection on the *cb* (**a**) and *ab* (**b**) planes of the helix formed in the **BenImi** crystal structure at 300 K. *Sh*_*h*_ helix pitch, *a*_*h*_ semi-major axis, *b*_*h*_ semi-minor axis.

The helix pitch value (Sh_h_) was determined along the *b* direction, which is associated with negative temperature expansion (see Fig. 2a). The *c* and *a* directions, associated with positive temperature expansion (Fig. 2a), were associated with the semi-major axis (*a*_h_) and semi-minor axis (*b*_h_) of the ellipse, respectively. For the **BenIm** salt, an analysis of temperature- and pressure-induced changes in helix parameters was carried out (Fig. 4, Table S8). With decreasing temperature/increasing pressure, there is an increase in the value of the helix pitch and a decrease in the value of the semi-major axis. In addition, we observe an increase in the value of the semi-minor axis with decreasing temperature/increasing pressure. The total temperature-dependent change in the parameter *a* of an elementary cell is affected by changes in the semi-minor axis (*b*_h_) and changes in the distance between the helixes (see Fig. S7).

**Figure 5:**
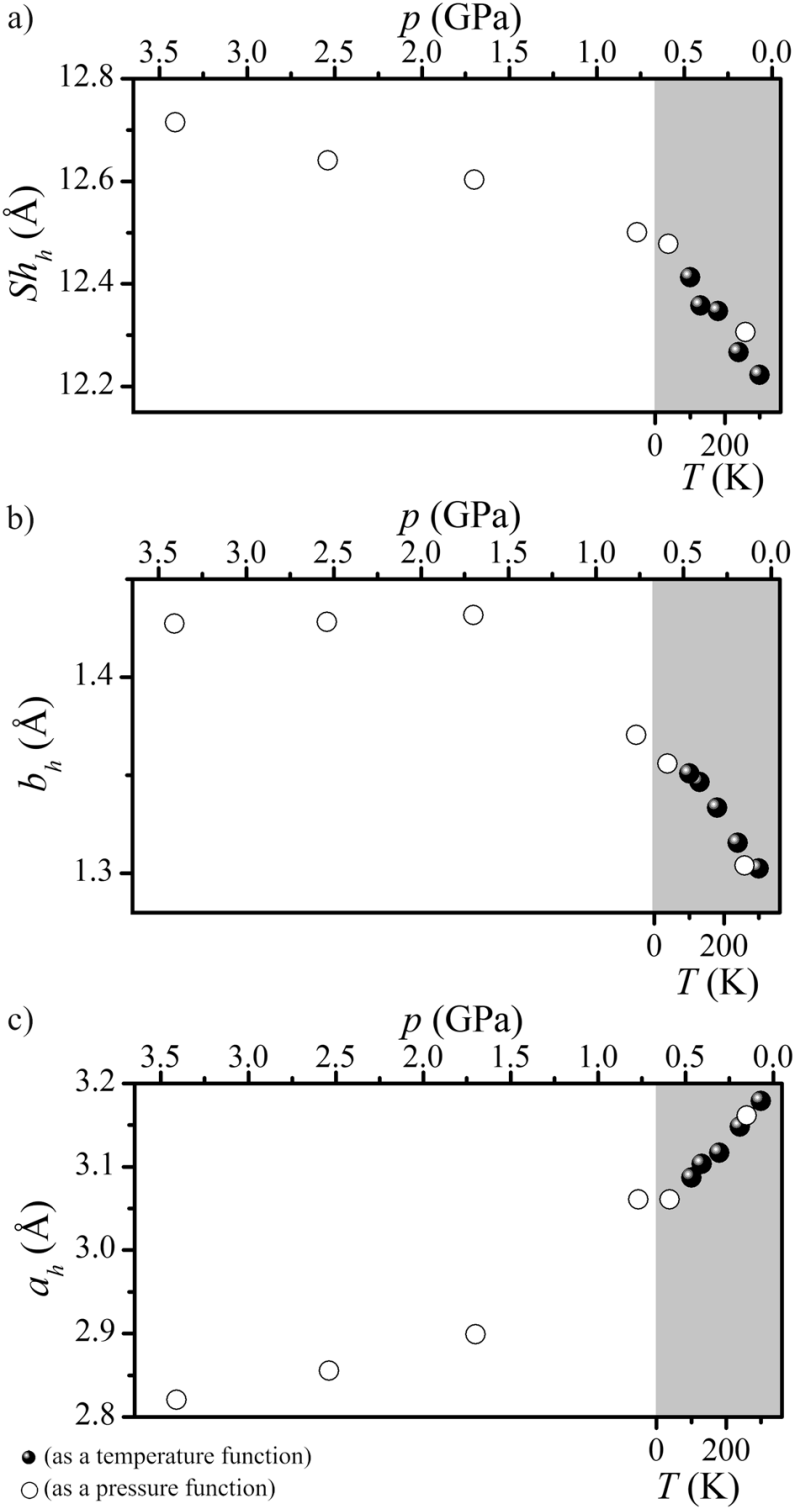
Dependence as a function of temperature and pressure of the helix pitch (**a**), the semi-minor axis (**b**), and the semi-major axis value (**c**).

In **BenImi**, a helical model can explain anomalous temperature and pressure expansion. As the temperature decreases, the semi-major axis value decreases while the helix pitch and semi-minor axis increase (Fig. 5). The decrease in the value of the semi-minor axis and the increase in the helix pitch with decreasing temperature/increasing pressure is a consequence of changes in the structure of the N^+^–H⋯O^−^ hydrogen bond network and the libration motion of imidazolium ions (Fig. S8). Imidazole ions rotate around an axis passing through C2–C3 carbon atoms. In addition to changes in helix parameters, the angle between the carboxylate group and the phenyl ring also changes (Fig. S8). It shows that the system moving to a lower energy structure “makes changes” not only in the dimensions of the helix itself but also in its internal structure.

**Figure 6:**
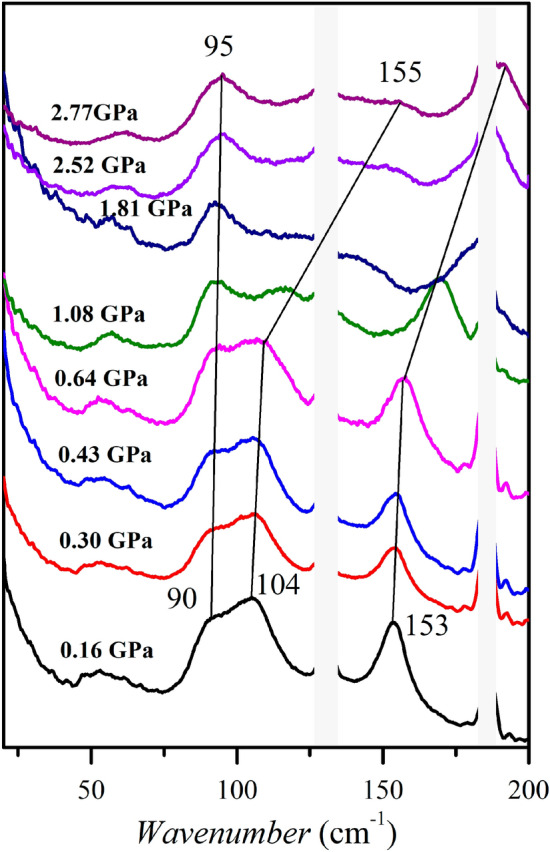
Raman spectrum of the **BenImi** salt as a function of hydrostatic pressure.

We can study the dynamics of hydrogen bond networks using absorption spectra and Raman scattering methods^47–49^. Bands below 200 cm^−1^ are associated with translational and librational network mods of the **BenImi** salt (Fig. 6). According to the X-Ray data, the lattice modes necessarily include the N^+^–H⋯O^−^ hydrogen bonds motions. Two features at about 160 and 100 cm^−1^ are related to hydrogen-bond stretching vibrations, and the other at about 50 cm^−1^ is the twisting vibrations^47–50^. As the pressure increases, the bands observed at about 160 and 100 cm^−1^ shift toward higher wavenumbers, indicating a shortening of hydrogen bond lengths, which is consistent with the results of the structural data (see Table S4).

**Figure 7:**
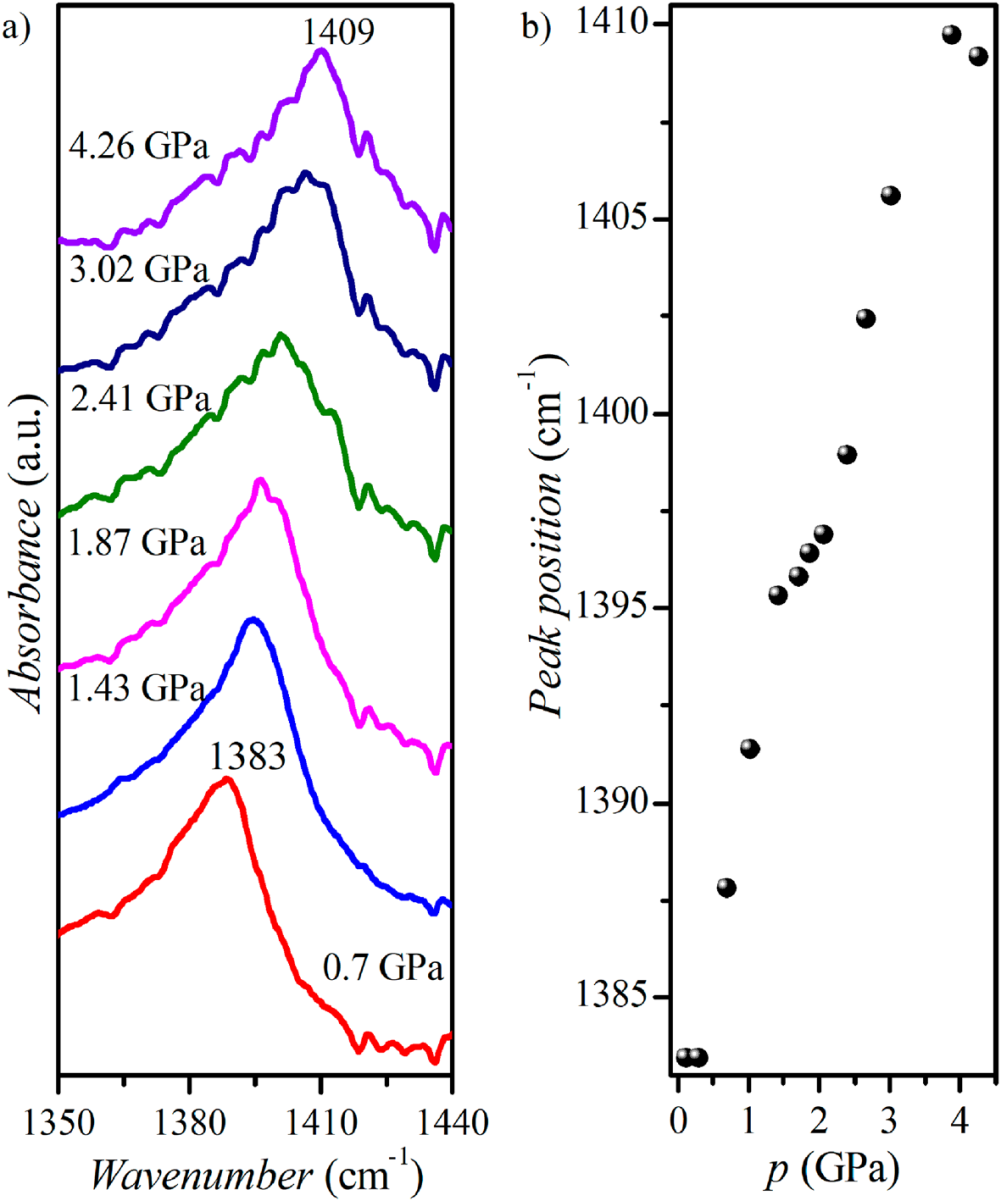
Absorbance spectrum of **BenImi** salt as a function of hydrostatic pressure (left) and the dependence of the position of the 1385 cm^−1^ band as a function of pressure (right).

Table S4 shows that as the pressure increases, the hydrogen bond angle of N^+^–H⋯O^−^ decreases. The dependence of the 1385 cm^−1^ band, related to *ν*_sym_(COO^−^)^37^, observed in the mid-infrared spectrum as a function of hydrostatic pressure (see Fig. 7) is consistent with these data. This band, observed in the absorption spectrum, is associated with the bending of the hydrogen bridge of N^+^–H⋯O^−^. In addition, spectroscopic studies revealed an anomaly within the 1–2 GPa region, confirming its occurrence in structural studies as a function of pressure (Fig. 7).

**Figure 8:**
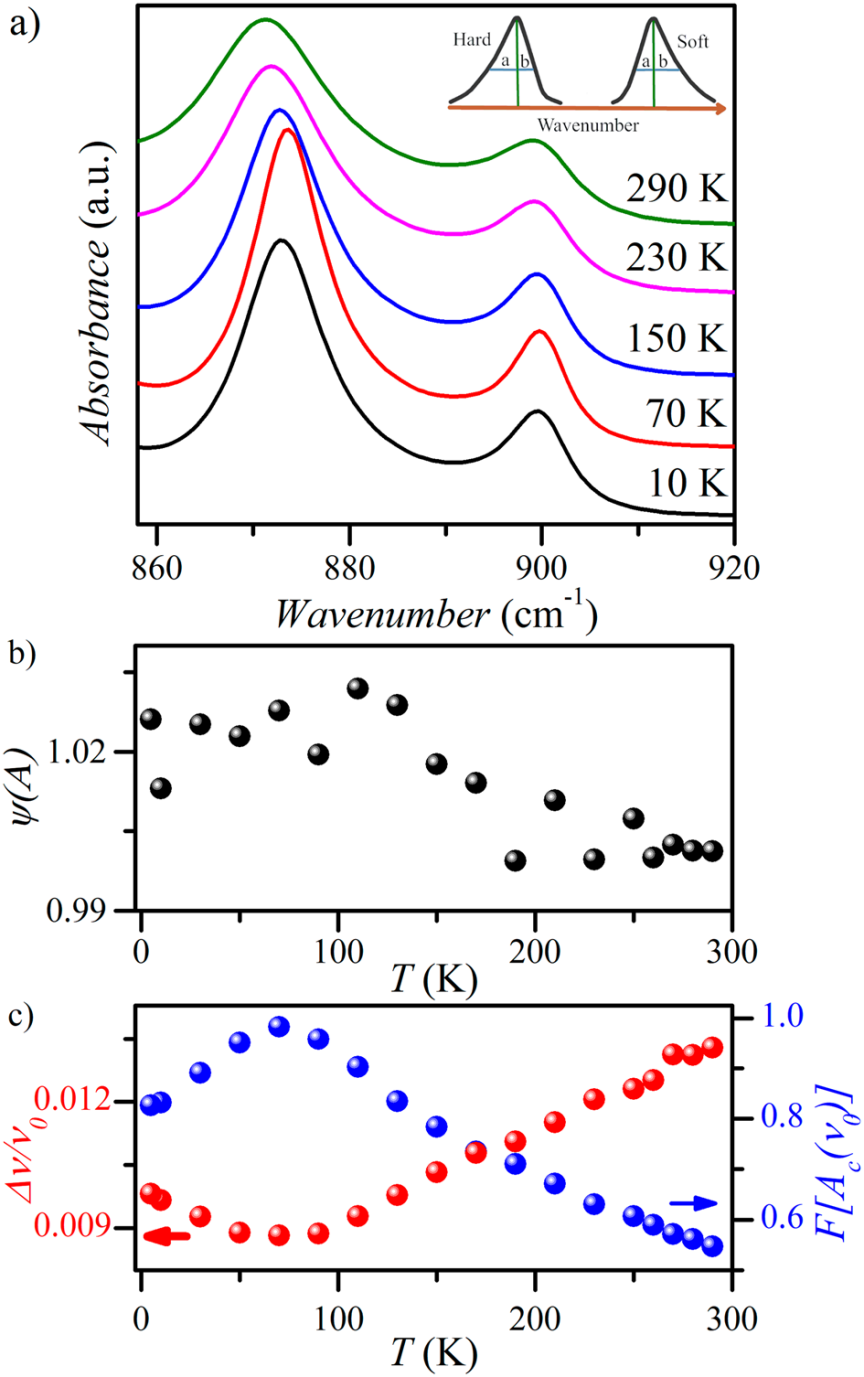
Infrared spectra of BenImi salt (**a**); anharmonicity coefficient (**b**), attenuation coefficient, and band intensity as a function of temperature (**c**).

From infrared spectroscopy studies, we can also determine whether the lattice vibrations involving the hydrogen bond network are harmonic or anharmonic^51,52^. It is possible by analyzing the absorption band's shape associated with the hydrogen bridges’ transverse vibrations^51^. In the case of anharmonic behavior, the band is asymmetric in which one slope is distinguished by a gentler slope directed toward a smaller value of the wavenumber (so-called “hard”) or a larger value of the wavenumber (so-called “soft”). The band is symmetric for harmonic behavior. We can describe the bands with parameters such as the damping parameter $\Delta \nu /\nu_{0}$ and the anharmonicity factor Ψ(A)^51^. We can calculate the anharmonicity factor using the determined value of *a* and *b* (see insert in Fig. 8a):

3 $$\Psi \left( A \right) = 1 + \frac{1}{\pi }\left[ {1 - \left( \frac{b}{a} \right)} \right],\quad \text{``for\;hard ''},$$

4 $$\Psi (A) = 1 - \frac{1}{\pi }\left[ {1 - \left( \frac{b}{a} \right)} \right],\quad \text{`` for\;soft''}.$$

Figure 8 shows the infrared spectra of **BenImi** salts recorded at 290, 230, 150, 70, and 10 K. The band near 870 cm^−1^ is associated with deformation vibrations of the N–H bond in the N^+^–H⋯O^−^ hydrogen bond^37^. As the temperature decreases, the intensity of the band increases, and its bandwidth decreases (Fig. 8c). Analysis of the band shape showed that it is characterized by “soft” (a < b) anharmonicity^51^. Figure 8b shows the dependence of the anharmonicity coefficient for **BenImi** as a function of temperature. A value of this coefficient greater than 1 introduces a negative contribution to the Grüneisen function, which leads to negative thermal expansion^51,52^.

## Conclusions

We have proposed a helical model to explain the negative linear thermal expansion an compression of imidazolium benzoate crystals. The mechanism of the negative linear behavior can be correlated with the structural distortions of helices. As the temperature drops and the hydrostatic pressure rises, the values of the parameters *a*, *c*, and *V* decrease while the value of parameter *b* rises. In the crystal lattice, ions interact by conventional N^+^–H⋯O^−^ (within helixes; medium strength) and unconventional C–H⋯O (between helixes; weak strength) hydrogen bonds. The interactions within the helix are stronger than interactions between helices. In a helical system with decreasing temperature and increasing pressure, the helix pitch increases while the value of the semi-major axis decreases. It leads to the elongation and contraction of the helix along its axis and in the perpendicular direction, respectively. These NLE and NLC effects have been correlated with the internal structure of the helix, involving the rotations of the imidazolium ions and rearrangements of the carboxylate groups.

## Methods

Imidazole benzoate salt (**BenImi**) was obtained by reacting benzoic acid with imidazole in the organic solvent ethyl acetate (99.8% purity, Merc KGaA)^37^. The substrates were weighed in a molar ratio 1:1. Each substrate was independently dissolved in ethyl acetate. Then the imidazole solution was added to the acid solution and stirred using a magnetic stirrer. The resulting white salt precipitate was drained on a Buchner funnel and washed with ethyl acetate to remove unreacted substrates.

Crystals of imidazolium benzoate (**BenImi**) suitable for single-crystal X-ray diffraction analysis were obtained by slow evaporation from solvent in ethyl acetate. X-ray diffraction measurements were carried out at 100, 130^37^, 180, 240, and 300 K on a SuperNova Dual Atlas diffractometer using Mo *Kα* radiation (λ = 0.71073 Å)^53^ (Table S1). The crystal structure was solved by the dual-space direct methods algorithm (SHELXT^54–56^) and refined against *F*^2^ using all data (SHELXL^55–57^). The positions of the H atoms bonded to the N atoms were obtained from difference Fourier maps and were refined freely with isotropic displacement parameters. The remaining H atoms were placed geometrically at calculated positions and refined using the riding model, with C–H=0.95 Å (C*sp*^2^H) for 100, 130^37^, 180 K or with C–H=0.94 and 0.93 Å (C*sp*^2^H) for 240 and 300 K, respectively and *U*_iso_(H) = 1.2*U*_eq_(C) for all measurement temperatures. The crystallographic data are available from the Cambridge Crystallographic Database Centre (CCDC 2265522 (100 K), CCDC 1892005 (130 K)^37^, 2265523 (180 K), 2265524 (240 K), 2265525 (300 K)). Molecular illustrations were prepared using ORTEP-3 for Windows^56^. The material for publication was prepared using WINGX^56^ and PLATON^58^.

High-pressure experiments were carried out with a modified Merrill–Bassett diamond-anvil cell (DAC)^59^. The pressure in the DAC chamber was calibrated by the ruby fluorescence with a Photon Control spectrometer, affording an accuracy of 0.02 GPa^60^. A good quality single crystal was placed in the DAC chamber with several ruby chips and the Daphne oil 7575 was used as the pressure-transmitting medium^61^. The pressure was gradually increased and several X-ray diffraction data sets were collected up to 3.41 GPa on a 4-cycle Excalibur EOS diffractometer (λ_MoKα_ = 0.71073 Å). Preliminary data reduction were performed with the CrysAlis software from Oxford Diffraction. The crystal structures of BenImi under high-pressure were solved by direct methods^54^ and refined by full-matrix least squares on F2’s^57^. The detail crystal data of high-pressure measurements are listed in Table S2 and also deposited in the CIF format with numbers CCDC 2263553–2263558.

Absorption spectra in the KBr matrix (*c* = 1:500), in the mid-infrared range (650–4000 cm^−1^), were recorded on a Bruker Equinox 55 FT-IR spectrometer coupled to a Hyperion 2000 microscope. They were recorded with a spectral resolution of 2 cm^−1^. A tungsten lamp was used as the radiation source. DLaTGS (400–7000 cm^−1^) and liquid nitrogen-cooled MCT (500–7000 cm^−1^) detectors were used. KBrGe was used as a beamsplitter. Studies of spectroscopic properties as a function of temperature were carried out using cryostats: Oxford Inst. CF 2102 (from 5 to 300 K) and Linkam THMS 600 (from 273 to 600 K). Infrared spectra as a function of pressure were recorded for **BenImi** using a Merrill–Bessett high-pressure chamber. Gasket Stainless was used as seals between the diamonds. Potassium bromide (BeCu) was used as a medium to transfer hydrostatic pressure from the anvil pistons to the sample. Ruby crystals were used to determine the value of the applied pressure. Type IIa diamonds (culet size—0.8 mm) embedded in anvil rings of BeCu were used.

Raman spectra were recorded on a LabRAM HR 800 UV HORIBA Jobin Yvon spectrometer, which is equipped with a liquid nitrogen-cooled CCD detector. The spectrum was recorded using He–Ne laser excitation (λ = 632.8 nm) with a spectral resolution better than 2 cm^−1^. The laser power on the sample was less than 1 mW. Raman spectra of **BenImi** were recorded in the spectral range from 50 to 3250 cm^−1^ using a 50 × objective. Raman spectra as a function of pressure were recorded using a Diacell^®^ CryoDAC-Mega diamond anvil. Cesium iodide (CsI) was used as a medium to transfer hydrostatic pressure from the anvil pistons to the sample; gasket of stainless steel were used. Ruby crystals were used to determine the value of the applied pressure. Type Ia diamonds (culet size—0.7 mm) were embedded in BeCu anvil rings.

### Supplementary Information

Supplementary Information.

## Data Availability

All data generated or analysed during this study are included in this published article (and its Supplementary Information files). The detail crystal data of low-temperature and high-pressure measurements are deposited in the CIF format, available free of charge on request form Cambridge Crystallographic Database Centre at https://www.ccdc.cam.ac.uk/structures/ with numbers: 1892005, 2265522–2265525 (low-temperature) and 2263553–2263558 (high-pressure).
