# Supplementary material for: Helical model of compression and thermal expansion
Source: Sci Rep. 2023 Oct 13;13:17398. doi: 10.1038/s41598-023-44467-y (PMC10575930; doi:10.1038/s41598-023-44467-y)
Supplement: Supplementary file 1 — Supplementary Information. [file 41598_2023_44467_MOESM1_ESM.pdf]

Helical Model of Compression and Thermal Expansion

Sylwia Zięba[1*], Michalina Rusek[2], Andrzej Katrusiak[2], Andrzej Gzella[3], Alina T. Dubis[4], and Andrzej Łapiński[1*]

[1]Institute of Molecular Physics, Polish Academy of Sciences, Smoluchowskiego 17, 60-179 Poznan, Poland
[2]Faculty of Chemistry, Adam Mickiewicz University, Uniwersytetu Poznańskiego 8, 61-614 Poznan, Poland
[3]Department of Organic Chemistry, Poznan University of Medical Sciences, Grunwaldzka 6, 60-780 Poznan, Poland
[4]Faculty of Chemistry, University of Bialystok, Ciołkowskiego 1K, 15-245 Bialystok, Poland

***e-mail: lapinski@ifmpan.poznan.pl; sylwia.zieba@ifmpan.poznan.pl**

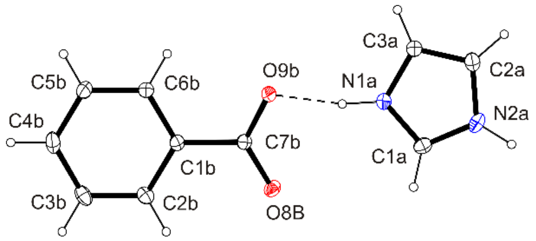

Figure S1. ORTEP view of the molecules of salt **BenImi** (100 K), showing the atomic labeling schemes. Non-H atoms are drawn as 30% probability displacement ellipsoids, and H atoms are drawn as spheres of arbitrary size.

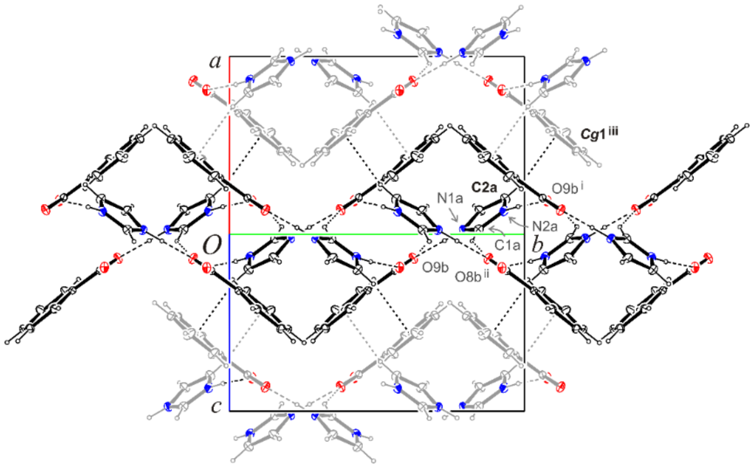

Figure S2. View of the unit cell of **BenImi** showing the layers of molecules parallel to the ($\bar{1}$01) plane. Adjacent layers are shown in black and gray for clarity of drawing. The symmetry codes are explained in Table S2.

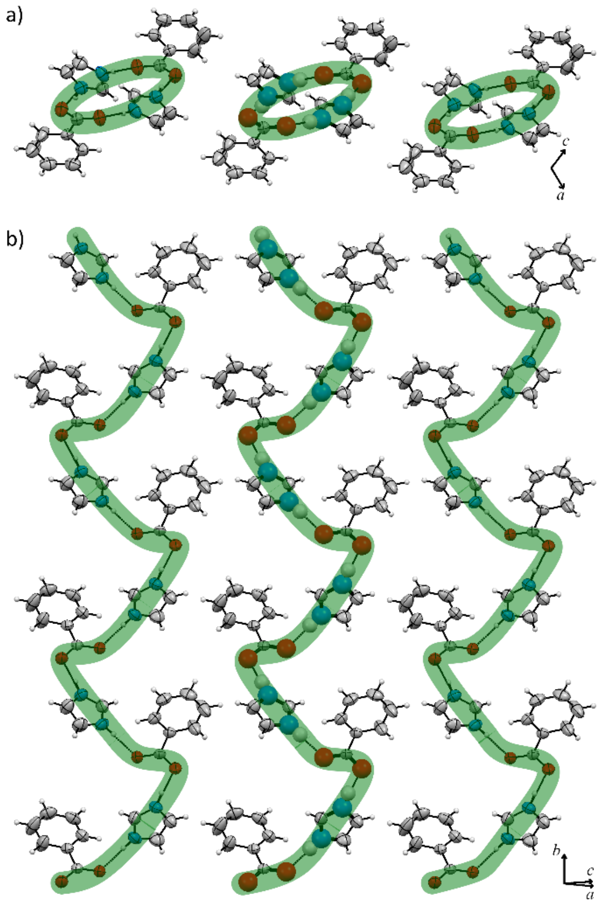

Figure S3. The helical structure in **BenImi** formed by anions and cations connected by N–H···O hydrogen bonds (shown in green).

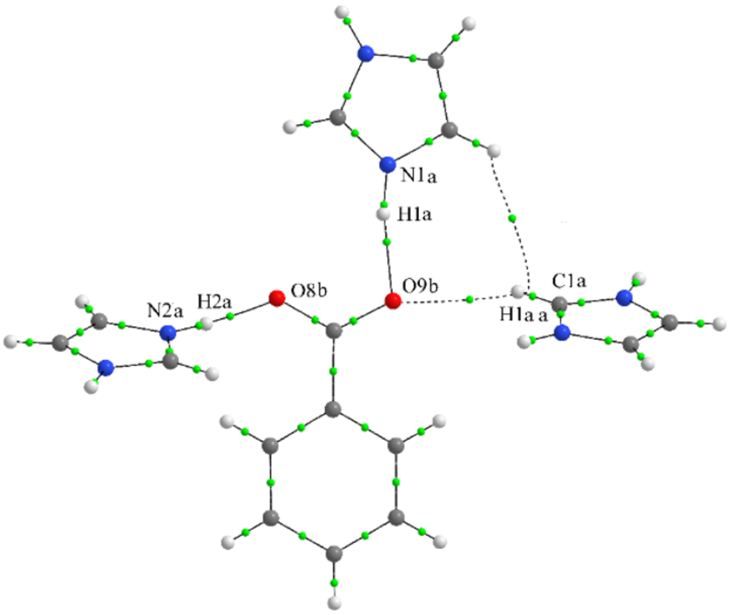

Figure S4. A room-temperature ion system at atmospheric pressure in BenImi, for which calculations were performed using QTAiM. The green dots correspond to bond critical points (BCPs).

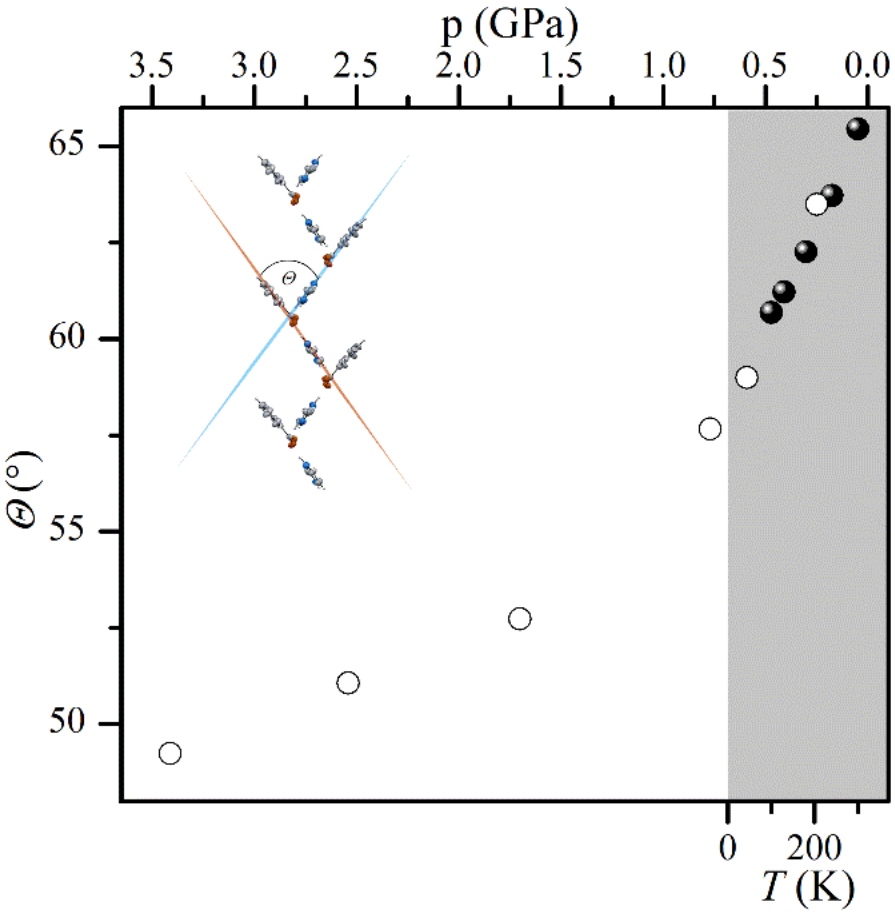

Figure S5. Change in the angle between the anion and cation plane (θ) as a function of temperature (solid circles) and pressure (open circles).

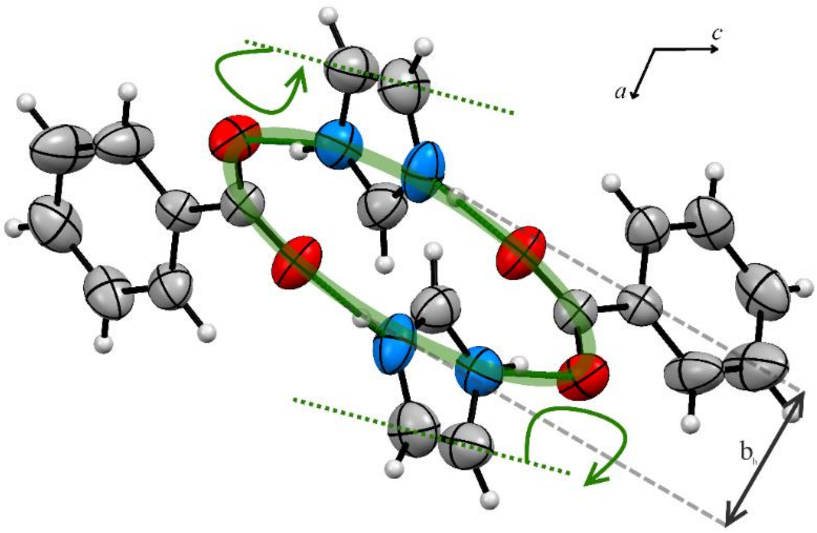

Figure S6. Librational motion of imidazole ion in **BenImi** salt.

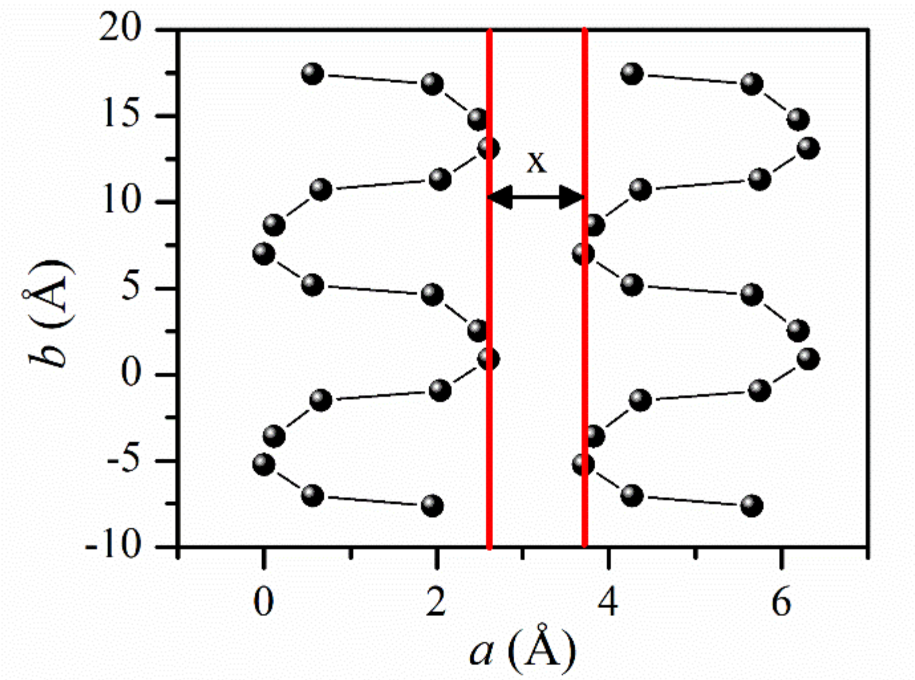

Figure S7. Projection on the *ab* planes of the helix formed in the **BenImi** crystal structure at 300 K. Note: The total temperature-dependent change in the parameter a of an elementary cell is affected by changes in the semi-minor axis (b_h) and changes in the distance between the helixes (denoted by X). The X value equals 1.097 Å (300 K) and 0.692 Å (100 K). The changes in this temperature range are ΔX=0.40 Å and Δb_h= 0.05 Å.

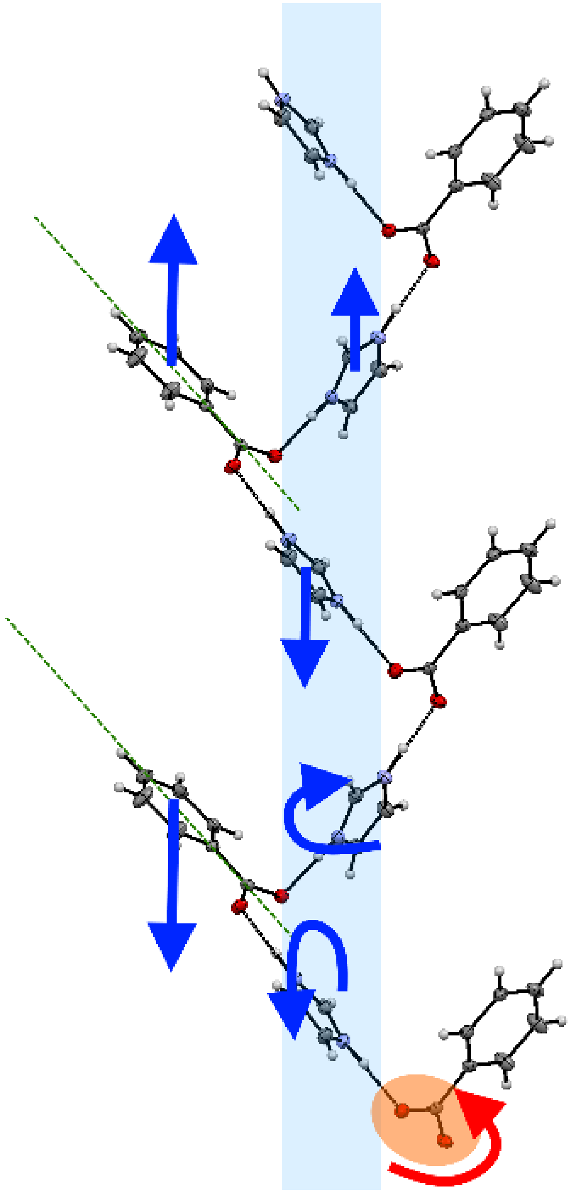

Figure S8. The hydrogen bond network is shown in plan view (-8.62, -0.05, 2.12). Note: The blue arrows show the direction of ion movement as the temperature decreases, whereas the red arrow shows the movement of the carboxylate group.

Table S1. Crystal data, data collection, and structure refinement for **BenImi** at different temperatures at atmospheric pressure

Crystal data					
Chemical formula	$C_{10}H_{10}N_2O_2$	$C_{10}H_{10}N_2O_2$	$C_{10}H_{10}N_2O_2$	$C_{10}H_{10}N_2O_2$	$C_{10}H_{10}N_2O_2$
M_r	190.20	190.20	190.20	190.20	190.20
Crystal system, space group			Monoclinic, $P2_1/n$		
Temperatur (K)	100.0(1)	130.0(1)	180.0(1)	240.0(1)	300.0(1)
Unit cell parameters					
a (Å)	8.6783(5)	8.7106(6)	8.7597(6)	8.8340(6)	8.9196(8)
b (Å)	12.3689(5)	12.3618(6)	12.3278(6)	12.2856(6)	12.2370(7)
c (Å)	9.2391(6)	9.2744(7)	9.3337(7)	9.4286(6)	9.5299(8)
β (deg)	111.397(7)	111.594(8)	111.930(9)	112.394(8)	112.890(10)
V (Å^3)	923.38(10)	928.56(12)	935.00(12)	946.13(12)	958.27(15)
Z	4	4	4	4	4
F(000)	400	400	400	400	400
D_x (Mg m^{-3})	1.368	1.361	1.351	1.335	1.318
Radiation type, λ(Å)			Mo $K\alpha$, 0.71073		
μ (mm^{-1})	0.098	0.097	0.096	0.095	0.094
Crystal shape			Block		
Colour			Colourless		
Crystal size (mm)			0.31 x 0.22 x 0.14		
Data collection					
Diffractometer	AuperNova, Dual Mo, Atlas	AuperNova, Dual Mo, Atlas	AuperNova, Dual Mo, Atlas	AuperNova, Dual Mo, Atlas	AuperNova, Dual Mo, Atlas
Absorption correction	Multi-scan (CrysAlis PRO). Empirical absorption correction using spherical harmonics, implemented in SCALE3 ABSPACK scaling algorithm.				
T_{min}, T_{max}	0.589, 1.000	0.655, 1.000	0.687, 1.000	0.605, 1.000	0.686, 1.000
No. of measured, independent and observed [$I > 2\sigma(I)$] reflections	10436, 2361, 2072	10482, 2348, 2008	10591, 2361, 1931	10689, 2405, 1942	10917, 2446, 1854
Measurement method			ω scans		
R_{int}	0.0364	0.0347	0.0332	0.0274	0.0283
θ_{max}, θ_{min} (º)	29.69, 2.76	29.68, 2.88	29.62, 2.88	29.58, 3.00	29.58, 2.99
Structure refinement					
R [$F^2 \geq 2\sigma(F^2)$], $wR(F^2)$, S	0.040, 0.1065, 1.086	0.042, 0.110, 1.059,	0.0449, 0.1189, 1.102	0.0479, 0.1360, 1.083	0.0549, 0.1682, 1.085
No. of reflections	2361	2348	2361	2405	2446
No. of parameters	135	135	135	135	135
$\Delta\rho_{max}$, $\Delta\rho_{min}$ (e Å^{-3})	0.322, −0.335	0.328, −0.275	0.254, −0.291	0.191, −0.283	0.218/, −0.270

Table S2. Crystal data, data collection, and structure refinement for imidazolium benzoate at different pressures at room temperature (*T*=293.02 K).

Chemical formula	$C_{10}H_{10}N_2O_2$	$C_{10}H_{10}N_2O_2$	$C_{10}H_{10}N_2O_2$	$C_{10}H_{10}N_2O_2$	$C_{10}H_{10}N_2O_2$	$C_{10}H_{10}N_2O_2$
Formula weight	190.20	190.20	190.20	190.20	190.20	190.20
Pressure (GPa)	0.25	0.59	0.77	1.70	2.54	3.41
Wavelength (Å)	0.71073	0.71073	0.71073	0.71073	0.71073	0.71073
Crystal system	Monoclinic	Monoclinic	Monoclinic	Monoclinic	Monoclinic	Monoclinic
Space group	$P2_1/n$	$P2_1/n$	$P2_1/n$	$P2_1/n$	$P2_1/n$	$P2_1/n$
Unit cell parameters (Å, º)	a=8.8389(10)<br>b=12.3031(14)<br>c=9.409(3)<br>β=112.260(19)	a=8.6065(7)<br>b=12.4147(12)<br>c=9.086(2)<br>β=111.014(17)	a=8.5466(11)<br>b=12.4409(17)<br>c=8.984(3)<br>β=110.62(2)	a=8.2252(14)<br>b=12.502(3)<br>c=8.509(6)<br>β=108.62(4)	a=8.1146(7)<br>b=12.5166(11)<br>c=8.365(3)<br>β=107.925(19)	a=8.0091(10)<br>b=12.5488(12)<br>c=8.205(4)<br>β=107.13(3)
Volume (Å^3)	946.9(3)	906.2(3)	894.1(4)	829.2(6)	808.3(3)	788.1(4)
Z	4	4	4	4	4	4
F(000)	400	400	400	400	400	400
Calculated density (g cm^{-3})	1.334	1.394	1.413	1.524	1.563	1.603
Absorption coefficient (mm^{-1})	0.095	0.100	0.101	0.109	0.112	0.114
Crystal dimensions [mm]	0.38 × 0.3 × 0.21					
Colour/Shape	Colourless/block					
Measurement method	Full-matrix least-squares on F^2					
Θ Range for data collection (º)	4.922 to 26.505	5.048 to 26.425	5.084 to 26.480	5.232 to 26.673	5.282 to 26.609	5.328 to 26.612
Max./Min. indices	h:-10→10<br>k:-13→13<br>l:-6→6	h:-10→10<br>k:-14→14<br>l:-6→6	h:-10→10<br>k:-14→14<br>l:-6→6	h:-9→9<br>k:-14→14<br>l:-5→5	h:-9→9<br>k:-14→14<br>l:-5→5	h:-9→9<br>k:-15→15<br>l:-4→4
Ref. collected/unique	4698/775	4416/726	4303/707	3888/649	3843/623	3696/578
Observed reflections [*I* ≥ 2□(*I*)]	1354	1343	1259	1177	1303	1148
R_{int}	0.0449	0.0470	0.0482	0.0542	0.0557	0.0513
Parameters	168	167	167	167	167	167
final R indices [$I>2\sigma(I)$]	R_1=0.0340<br>wR_2=0.0767	R_1=0.0346<br>wR_2=0.0723	R_1=0.0361<br>wR_2=0.0810	R_1=0.0359<br>wR_2=0.0733	R_1=0.0334<br>wR_2=0.0695	R_1= 0.0396<br>wR_2= 0.0753
R indices (all data)	R_1=0.0684<br>wR_2=0.0921	R_1=0.0639<br>wR_2=0.0844	R_1=0.0669<br>wR_2=0.0966	R_1=0.0663<br>wR_2=0.0847	R_1=0.0605<br>wR_2=0.0808	R_1= 0.0701<br>wR_2= 0.0873
goodness of fit of F^2	1.051	1.087	1.073	1.079	1.063	1.168
Largest diff. peak and hole	0.07/-0.07	0.08/-0.09	0.10/-0.11	0.11/-0.10	0.10/-0.10	0.10/-0.10

Table S3. Hydrogen-bond geometry (Å, °) for **BenImi** at different temperatures.

Temperature (K)	*D*—H···*A*	*D*—H	H···*A*	*D*···*A*	*D*—H···*A*
100	N1a–H1a···O9b<br>N2a–H2a···O8b^{i}<br>C1a–H1aa···O9b^{ii}<br>C3a–H3aa···*Cg*1^{iii}	0.951(18)	1.660(18)	2.6009(13)	169.4(18)
		0.914(18)	1.736(17)	2.6469(13)	174.63(15)
		0.95	2.38	3.2038(16)	145
		0.95	2.64	3.5316(14)	156
130*		0.94(2)	1.67(29)	2.6033(14)	168.4(16)
		0.922(18)	1.730(18)	2.6495(14)	174.6(18)
		0.95	2.38	3.2032(17)	145
		0.95	2.66	3.5531(16)	156
180		0.98(2)	1.64(2)	2.6036(15)	168.1(18)
		0.912(19)	1.743(19)	2.6521(16)	174.2(18)
		0.95	2.38	3.1981(19)	144
		0.95	2.69	3.5880(18)	157
240		1.02(2)	1.60(2)	2.6085(16)	170(3)
		0.91(3)	1.75(2)	2.6576(17)	175(3)
		0.94	2.39	3.196(2)	144
		0.94	2.74	3.636(2)	159
300		0.97(3)	1.66(3)	2.614(2)	168(3)
		0.84(3)	1.83(3)	2.668(2)	176(2)
		0.93	2.39	3.191(2)	144
		0.93	2.80	3.689(3)	160

Symmetry codes: (i) 1/2-*x*, 1/2+*y*, 1/2-*z*; (ii) -1/2+*x*,3/2-*y*,-1/2+*z*; (iii) 1/2+*x*,3/2-*y*,-1/2+z. *Cg*1 is a phenyl ring centroid.

Table S4. Hydrogen-bond geometry (Å, °) for **BenImi** at different pressures.

<table>
<tr><th>Pressure (GPa)</th><th>D—H···A</th><th>D—H</th><th>H···A</th><th>D···A</th><th>D—H···A</th></tr>
<tr><td rowspan="3">0.25</td><td rowspan="18">N1a–H1a···O9b
N2a–H2a···O8b^{i}
C1a–H1aa···O9b^{ii}</td><td>0.97(2)</td><td>1.67(5)</td><td>2.621(4)</td><td>163(4)</td></tr>
<tr><td>0.93(3)</td><td>1.74(3)</td><td>2.665(4)</td><td>178(3)</td></tr>
<tr><td>0.85(2)</td><td>2.46(2)</td><td>3.194(4)</td><td>144(2)</td></tr>
<tr><td rowspan="3">0.59</td><td>1.01(5)</td><td>1.61(5)</td><td>2.612(4)</td><td>170(3)</td></tr>
<tr><td>0.98(4)</td><td>1.68(4)</td><td>2.656(4)</td><td>173(4)</td></tr>
<tr><td>0.88(2)</td><td>2.40(2)</td><td>3.176(4)</td><td>148(2)</td></tr>
<tr><td rowspan="3">0.77</td><td>0.91(6)</td><td>1.72(7)</td><td>2.615(4)</td><td>166(6)</td></tr>
<tr><td>0.98(4)</td><td>1.68(4)</td><td>2.656(4)</td><td>177(5)</td></tr>
<tr><td>0.88(3)</td><td>2.41(3)</td><td>3.168(4)</td><td>146(3)</td></tr>
<tr><td rowspan="3">1.70</td><td>1.04(6)</td><td>1.59(7)</td><td>2.592(4)</td><td>160(6)</td></tr>
<tr><td>0.93(4)</td><td>1.70(4)</td><td>2.629(4)</td><td>175(4)</td></tr>
<tr><td>0.89(2)</td><td>2.32(2)</td><td>3.122(4)</td><td>150(2)</td></tr>
<tr><td rowspan="3">2.64</td><td>1.01(6)</td><td>1.62(7)</td><td>2.603(4)</td><td>166(5)</td></tr>
<tr><td>0.95(3)</td><td>1.67(3)</td><td>2.617(4)</td><td>172(4)</td></tr>
<tr><td>0.95(3)</td><td>2.26(3)</td><td>3.106(3)</td><td>149(2)</td></tr>
<tr><td rowspan="3">3.42</td><td>0.92(6)</td><td>1.73(8)</td><td>2.599(5)</td><td>157(8)</td></tr>
<tr><td>0.96(4)</td><td>1.66(5)</td><td>2.611(5)</td><td>171(7)</td></tr>
<tr><td>0.94(4)</td><td>2.26(4)</td><td>3.087(5)</td><td>146(2)</td></tr>
</table>

Symmetry codes: (i) -1/2+*x*, 1/2-*y*, -1/2+*z*; (ii) 1-*x*, 1-*y*, 2-*z*; (iii) 1/2+*x*,3/2-*y*,-1/2+*z*.

Table S5. Cell parameters (Å, °) for **BenImi** at different temperatures.

Temperature (K)	*a*	*b*	*c*	*α*	*β*	*γ*	*V*
100	8.6783(5)	12.3689(5)	9.2391(6)	90.00	111.397(7)	90.00	923.38(10)
130*	8.7106(6)	12.3618(6)	9.2744(7)	90.00	111.594(8)	90.00	928.56(12)
180	8.7597(6)	12.3278(6)	9.3337(7)	90.00	111.930(9)	90.00	935.00(12)
240	8.8340(6)	12.2856(6)	9.4286(6)	90.00	112.394(8)	90.00	946.13(12)
300	8.9196(8)	12.2370(7)	9.5299(8)	90.00	112.890(10)	90.00	958.27(15)

Table S6. QTAiM parameters (in atomic unit) corresponding to the H···O bond critical point (BCPs), the electron density at BCP ρ_{BCP}; Laplacian of electron density at BCP, $\Delta^2\rho_{BCP}$; total electron energy density at BCP, H_{BCP} and the components of the H_{BCP}: kinetic electron energy density, G_{BCP}; potential electron energy density, V_{BCP}, the hydrogen bond energy, E_{HB}.

	T (K)	ρ_{BCP}	Δ_{BCP}	G_{BCP}	V_{BCP}	H_{BCP}	E_{HB}
N1a–H1a···O9b	300	0.0537	0.1508	0.0457	-0.0538	-0.0081	-16.88
	240	0.0605	0.1485	0.0495	-0.0619	-0.0124	-19.41
	180	0.0429	0.1572	0.0406	-0.0419	-0.0013	-13.17
	130	0.0509	0.1569	0.0453	-0.0513	-0.0060	-16.09
	100	0.0524	0.1573	0.0462	-0.0531	-0.0069	-16.65
N2a–H2a···O8b	300	0.0325	0.1402	0.0322	-0.0295	0.0028	-9.24
	240	0.0394	0.1480	0.0372	-0.0374	-0.0002	-11.72
	180	0.0372	0.1477	0.0359	-0.0350	0.0009	-10.99
	130	0.0415	0.1503	0.0387	-0.0399	-0.0012	-12.53
	100	0.0377	0.1496	0.0365	-0.0357	0.0008	-11.20
C1a–H1aa···O9b	300	0.0098	0.0372	0.0076	-0.0058	0.0018	-1.82
	240	0.0099	0.0374	0.0076	-0.0059	0.0018	-1.84
	180	0.0101	0.0378	0.0077	-0.0059	0.0018	-1.87
	130	0.0100	0.0376	0.0077	-0.0059	0.0017	-1.85
	100	0.0100	0.0377	0.0077	-0.0059	0.0018	-1.86

Table S7. QTAiM parameters (in atomic unit) corresponding to the H···O bond critical point (BCPs), the electron density at BCP ρ_{BCP}; Laplacian of electron density at BCP, $\Delta^2\rho_{BCP}$; total electron energy density at BCP, H_{BCP} and the components of the H_{BCP}: kinetic electron energy density, G_{BCP}; potential electron energy density, V_{BCP}, the hydrogen bond energy, E_{HB}.

	p (GPa)	ρ_{BCP}	Δ_{BCP}	G_{BCP}	V_{BCP}	H_{BCP}	E_{HB}
N1a–H1a···O9b	0.15	0.0551	0.1461	0.0456	-0.0548	-0.0091	-17.18
	0.59	0.0576	0.1498	0.0479	-0.0584	-0.0105	-18.33
	0.77	0.0432	0.1514	0.0397	-0.0415	-0.0018	-13.02
	1.70	0.0619	0.1463	0.0498	-0.0631	-0.0133	-19.79
	2.64	0.0592	0.1463	0.0482	-0.0597	-0.0116	-18.74
	3.42	0.0505	0.1529	0.0441	-0.0499	-0.0058	-15.64
N2a–H2a···O8b	0.15	0.0339	0.1417	0.0333	-0.0311	0.0023	-9.75
	0.59	0.0348	0.1443	0.0341	-0.0321	0.0019	-10.08
	0.77	0.0348	0.1442	0.0341	-0.0321	0.0019	-10.08
	1.70	0.0372	0.1532	0.0368	-0.0354	0.0015	-11.09
	2.64	0.0385	0.1569	0.0382	-0.0371	0.0011	-11.64
	3.42	0.0389	0.1581	0.0386	-0.0377	0.0009	-11.82
C1a–H1aa···O9b	0.15	0.0098	0.0374	0.0076	-0.0058	0.0018	-1.83
	0.59	0.0102	0.0395	0.0080	-0.0061	0.0019	-1.92
	0.77	0.0104	0.0403	0.0082	-0.0062	0.0019	-1.96
	1.70	0.0114	0.0456	0.0092	-0.0070	0.0022	-2.19
	2.64	0.0117	0.0471	0.0095	-0.0072	0.0023	-2.27
	3.42	0.0122	0.0491	0.0099	-0.0076	0.0024	-2.37

Table S8. Parameters of the helicoid: a_h (semi-major axis), b_h (semi-minor axis), Sh_h (helix pitch), c_h (focal length of the ellipse), e_h (eccentricity of the ellipse), l_h (circumference of the ellipse), and S_h (area of the ellipse).

		2a (Å)	a (Å)	2b (Å)	b (Å)	Sh_h (Å)	c_h (Å)	e_h	S_h (Å^2)	l_h (Å)
T (K)	300	6.36	3.18	2.61	1.30	12.22	2.89	0.91	13.00	14.69
	240	6.29	3.15	2.63	1.32	12.27	2.86	0.91	13.00	14.61
	180	6.23	3.12	2.67	1.33	12.35	2.82	0.90	13.05	14.54
	130	6.21	3.10	2.69	1.35	12.36	2.79	0.90	13.12	14.52
	100	6.17	3.09	2.70	1.35	12.41	2.78	0.89	13.09	14.47
p (GPa)	0.15	6.32	3.16	2.61	1.30	12.31	2.88	0.91	12.94	14.63
	0.59	6.12	3.06	2.71	1.36	12.48	2.74	0.89	13.03	14.39
	0.77	6.12	3.06	2.74	1.37	12.50	2.74	0.89	13.17	14.43
	1.70	5.79	2.89	2.86	1.43	12.60	2.52	0.87	13.03	13.99
	2.54	5.71	2.86	2.86	1.43	12.64	2.47	0.87	12.80	13.83
	3.41	5.64	2.82	2.85	1.43	12.71	2.42	0.86	12.77	13.74
